# Maternal Nicotine Exposure During Gestation and Lactation Period Affects Behavior and Hippocampal Neurogenesis in Mouse Offspring

**DOI:** 10.3389/fphar.2019.01569

**Published:** 2020-01-22

**Authors:** Fei Liu, Xinrong Tao, Gang Pang, Diqing Wu, Yuting Hu, Song Xue, Jing Liu, Bing Li, Li Zhou, Qiang Liu, Yong-Mei Zhang

**Affiliations:** ^1^ Center for Medical Research, School of Medicine, Anhui University of Science and Technology, Huainan, China; ^2^ Key Laboratory of Industrial Dust Deep Reduction and Occupational Health and Safety of Anhui Higher Education Institutes, Anhui University of Science and Technology, Huainan, China; ^3^ Key Laboratory of Industrial Dust Purification and Occupational Health of the Ministry of Education, Anhui University of Science and Technology, Huainan, China; ^4^ College of Basic Medical Science, Anhui Medical University, Hefei, China; ^5^ The First Affiliated Hospital of Anhui University of Science and Technology, Huainan, China; ^6^ Jiangsu Province Key Laboratory of Anesthesiology, Xuzhou Medical University, Xuzhou, China

**Keywords:** maternal nicotine exposure, hippocampus, neurogenesis, mouse, offspring

## Abstract

Cigarette smoking or nicotine exposure during pregnancy is associated with numerous obstetrical, fetal, and developmental complications, as well as an increased risk of adverse health consequences in the adult offspring. In this study, we examined the effects of maternal nicotine exposure during perinatal and lactation stages on behavioral performance and hippocampal neurogenesis in the adolescent stage of offspring mice. Female C57BL/mice received nicotine in drinking water (200 μg/ml nicotine) or vehicle (1% saccharin) starting from 2 weeks premating until the offspring were weaned on postnatal day 20. Experiments started on postnatal day 35. Female offspring with maternal nicotine exposure presented an increase in anxiety-like behavior in an open-field test. BrdU assay revealed that nicotine offspring presented an increase in cell proliferation in hippocampal dentate gyrus, but the number of BrdU^+^ cells was decreased in one week and further decreased in three weeks. The occurrence of disarray of DCX^+^ cells increased in both male and female nicotine offspring. The density of microglial marker protein Iba1 was significantly increased in the nicotine offspring. Furthermore, the expression of microglia marker Iba1, the CX3CL1, CX3CR1, and downstream molecules PKA and p-ErK were significantly increased in the nicotine group. In summary, maternal nicotine exposure affects both hippocampal neurogenesis and microglial activity in the adolescent offspring.

## Introduction

Besides smokers, more than 40% of children, 33% of nonsmoking men and 35% of nonsmoking women are exposed to tobacco (Hernandez-Martinez et al., 2017). It has been well documented that cigarette smoking during pregnancy is associated with a number of adverse obstetrical outcomes and developmental issues in the offspring (Bruin et al., 2010). Direct or indirect exposure to tobacco smoke during pregnancy increases the risk for premature birth, rupture of membranes, intrauterine growth delay, and sudden neonatal death syndrome (Holbrook, 2016). Besides the negative effect on embryo and fetus development, maternal smoking exposure may induce long-term and delayed adverse effects on the cognitive development of nicotine offspring in childhood (Balazs et al., 2018). Moreover nicotine in lactate can be absorbed by offspring and causes behavioral irritation and anxiety in the offspring (Karimiankakolaki et al., 2019). Smoking cessation or at least reduction of cigarette smoking during pregnancy can ameliorate damage to the developing fetus (Lindley et al., 2000).

Neurogenesis is the process by which neural progenitor cells differentiate into new mature neurons (Rikani et al., 2013). Most neurogenesis occurs in the embryonic period, however, neurogenesis in the subgranular layer of the hippocampal dentate gyrus (DG) and the subventricular zone (SVZ) of the lateral ventricle (Ohishi et al., 2014) continues throughout the life cycle. Nicotine in tobacco is a neuroteratogen that can cross the placenta and alter the pattern of cell proliferation, differentiation, and myelin formation during embryo and fetus development by activating the acetylcholine receptors (AChRs). Studies have shown that self-administered nicotine reduces neurogenesis in the hippocampus, which in turn affects learning and memory in mice (Csabai et al., 2016). The role of maternal nicotine exposure on hippocampal neurogenesis in the adolescent offspring is not fully understood.

Microglia are the embryonic derived macrophages of self-renewing tissue in the central and peripheral nervous system (Song and Colonna, 2018). Microglia have a variety of functions, for example, supporting neuronal structures and maintaining homeostasis (Lannes et al., 2017). Also, microglial activation is involved in neuroinflammation, which may play a neuroprotective role by removing neurotoxic factors (Egea et al., 2015), or may cause neurotoxic damage through increased levels of proinflammatory mediators and oxidative stress (Aaseth et al., 2018; Allen and Lyons, 2018; Quik et al., 2019). Microglia regulate neuronal migration through the CX3CL1/CX3CR1 ligand receptor binding signaling pathway. Some studies have shown that prenatal nicotine exposure can cause changes in the number and morphology of microglia and increase the expression of cytokines in the cortex of developing mice (Kagawa and Nagao, 2018). However, the impact of maternal nicotine exposure on microglial function in the adolescent offspring remains elusive.

In this study, we examined the effects of maternal nicotine exposure during prepregnancy, pregnancy, and prolactation on the behaviors, hippocampal neurogenesis, and microglial activation in the adolescent stage of the offspring. Our data revealed that maternal nicotine exposure alters behavior, hippocampal neurogenesis, and microglial activation in the offspring.

## Materials and Methods

### Animals

Adult C57BL/6 mice were purchased from the Changzhou Cavion Experimental Animal Co, Ltd. (license number SCXY (Su) 2011-0003). Mice were housed in a vivarium maintained on a standard 12 h light–dark cycle (lights on at 07:00 AM), with constant temperature and humidity (22°C and 50%, respectively), and ad libitum access to food and water. All procedures were conducted in accordance with the guidelines as described in the National Institutes of Health’s Guide for the Care and Use of Laboratory Animals (NIH Publication No. 8023, revised 1978) and were approved by the Institutional Animal Care and Use Committee at Anhui University of Science and Technology.

### Maternal Nicotine Exposure

Female mice (n = 9) were administered nicotine (Sigma, N-3876; 200 μg/ml) in drinking water containing 1% saccharin (Shyuanye; 128-44-9) starting from 2 weeks premating until the offspring were weaned. The female control mice (n = 9) received drinking water containing 1% saccharin. Two weeks after drinking nicotine water, all female mice were paired with male mice at a 3:1 ratio until they gave birth. All female mice gave birth to babies. The offspring were weaned on postnatal day 20 (P20) and distributed randomly into experimental groups (**Supplementary Table 1**).

### 5-Bromo-2’-Deoxyuridine (BrdU) Staining

Offspring received an intraperitoneal injection of BrdU (Sigma, B5002; 10 mg/ml) on P35. For the proliferation study, male and female offspring (n > 3) were given two injections of BrdU (100 mg/kg, i.p.) at a 6-h interval. Twenty-four hours after BrdU administration, mice were deeply anesthetized and perfused transcardially with ice-cold PBS, followed by 4% paraformaldehyde (Sangon Biotech). Brains were harvest and fixed for 48 h in 4% paraformaldehyde, followed by 30% sucrose dehydration. Another cohort of offspring received BrdU (50 mg/kg, i.p.) once a day for five consecutive days to examine neuronal survival. Mice were sacrificed on day 7 (survival one week) and day 21 (survival three weeks) after the last injection of BrdU.

### Physical Development

The offspring were weighed at postnatal days 0, 5, 10, 15, 20, 25, 30, and 35 days. The pinna detachment was defined as bilateral ear protrusion in a completely upright state without any adhesion to the brain. Hair growth was determined as the day when white soft hair was observed on the whole body of the offspring. Incisor eruption was evaluated that two Incisors in the upper jaw were erupted and white gums with a clear boundary muscles were seen. Eye opening was based on the degree of binocular opening that was equal to or greater than 1/2 of normal palpebral fissure.

### Neurobehavioral Development

Righting reflex test started on P7. The litters were put in supine position. The time mice spent reverting to the prone position was recorded. The cliff avoidance test started on P7. Experiment was terminated if the forelimbs failed to complete within 30 s. The negative geotaxis test started on P7 by putting the offspring head down on a 25° nonsmooth slope, and recorded the time required for the offspring to turn 180° from head down to head up. Experiment stopped if the mouse failed to complete within 30 s. The air righting reflex test started on P8 for consecutive days by turning the offspring on its supine position and letting go of hands from 15 cm away from the bottom of the cage (with sawdust 5-cm thick). The posture of the offspring falling to the ground and the four-claw landing was recorded. The olfactory reflex test started on P12 for consecutive days by placing the offspring in the center of a box with a clean absorbent cotton on one side and a absorbent cotton with smell on the other side to evaluate whether the offspring could distinguish the smell of the cage and reach the side of the absorbent cotton with the smell. Auditory startle test started on P12 for consecutive days. The offspring were placed in a cage alone and a metal block in 15 cm away from the offspring was hit. The positive reaction includes body suddenly curl, arch, and two consecutive positive stimulus responses were defined as the standard. Forelimb grasp was tested on the 12th day after birth by putting the forelimb of the offspring on a wooden circular rod fixed in a horizontal position. The rod is about 0.2 cm in diameter and about 30 cm above the ground. Duration that the offspring griped the rod was recorded.

### Behavioral Tests

#### Open-Field Test

Open-field testing started at P35. Locomotor activity was tracked *via* an overhead video camera interfaced with behavioral tracking software EthoVision XT 5.1 (Noldus Information Technology, The Netherlands). After 3-day habituation to behavioral recording room for 60 min and arena for 10 min, mouse was gently placed in a center of an open-field Plexiglas clear chamber (30 cm × 30 cm × 35 cm) and allowed to move freely for 1 h. Zone within 7.5 cm away from the wall is considered peripheral area. The rest is central zone. All chambers were cleaned thoroughly with 10% ethanol between trials to remove odor residue. Distance traveled, defined as the sum of recorded movement of the center point of the mouse, in centimeter over the duration of the trial. Immobility, defined as the amount of time, in seconds, that Ethovision failed to detect any linear or angular movement of the animal. Immobility was determined by measuring the amount of change in pixels from one 3-frame sample to the next; if the total pixel area representing the mouse changed by less than 20%, then the mouse was considered to be immobile. A mouse that reared or was grooming would not be detected as immobile (Vick KAt and Stackman, 2010).

#### The Elevated Plus Maze Test

The elevated plus maze (EPM) was a test for measuring anxiety in rodents. The EPM test setting for the mice was an apparatus with two plus-shaped horizontal 45 cm × 5 cm lanes. At the crossing of the planes there was an open central 5 cm × 5 cm platform. The mice were placed in the behavioral laboratory about 3 h in advance to adapt to the environment and reduce the stress of the mice. The experiment was conducted in daylight (150–200 lx). The mice were initially removed from the cage, placed with their backs to the experimenter and their heads facing the open arms and placed head-first at the junction of the open and closed arms. Mice were allowed to move freely. The locomotor activity was captured by an overhead camera and analyzed by the Smart v2.5.21 software. Maze was cleaned with 75% ethanol between recordings.

#### Tail Suspension Test

Each mouse was suspended on the edge of a rod 50 cm above a tabletop using adhesive Scotch tape placed approximately 1 cm from the tip of the tail. The mice were hung for 6 min. The duration of immobility was measured and recorded by observers. The mice were considered immobile when they showed no body movement during the test.

### Immunofluorescence Staining

#### Preparation of Brain Slices

Hippocampus was sliced at coronal section at a thickness of 40 μm. Every 12th section was selected and processed to make a series of slices for staining and counting. The number of BrdU^+^ cells in the granule cell Layer (GCL) of the hippocampal DG in each series of sections were multiplied by 12 as an estimation of the total number of BrdU^+^ cells for the proliferation and survival studies (Salvi et al., 2016).

#### BrdU Staining

Brain slices were permeabilized with 1% Triton X-100 and 0.5% Tween 20 in PBS, followed by 1 N HCl for 10 min at room temperature, 2 N HCl for 10 min at room temperature and 20 min at 37°C to denature DNA. After Borate buffer (0.1 M) and TBS washing, the slices were blocked with 5% goat serum (Beyotime Biotechnology, C0265) for 1 h before being incubated in anti-BrdU antibody (**Table 1**) at 4°C overnight. After PBS washing, slices were incubated with Goat anti-Rat IgG H&L for 1.5 h at room temperature. DAPI (1:2000; Beyotime Biotechnology, 1002) was used for nucleus staining. Slices were mounted with Antifade Mounting Medium (Beyotime Biotechnology, P0126) and stored at 4°C in dark until image capture and analysis.

**Table 1:** Primary antibodies used in immuno-staining.

Antigen	Host	Dilution	Company
BrdU	Rat, monoclonal	1:40	abcam, ab6326
GFAP	Rabbit, polyclonal	1:500	abcam, ab7260
Iba1	Rabbit, polyclonal	1:500 (IF)	Wako, 019-19741
Iba1	Rabbit, monoclonal	1:1000 (WB)	abcam, ab178846
DCX	Rabbit, monoclonal	1:100	abcam, ab207175
CX3CL1	Rabbit, Polyclonal	1:1000	abcam, ab25088
CX3CR1	Rabbit, Polyclonal	1:500	Proteintech, 13885-1-AP
PKA	Rabbit, monoclonal	1:1000	Cell Signaling Technology, 5842
ErK	Rabbit, monoclonal	1:1000	Cell Signaling Technology, 4695
p-ErK	Rabbit, monoclonal	1:2000	Cell Signaling Technology, 4370
GAPDH	Rabbit, Polyclonal	1:10000	Proteintech, 10494-1-AP
Goat anti-Rat IgG H&L	Rat, Polyclonal	1:1000	Life Technology, A-11006
Goat anti-rabbit IgG H&L	Rabbit, polyclonal	1:1000	Life Technology, A-11037

#### BrdU and GFAP Double Immuno-Staining

Slices were incubated with anti-BrdU antibody and anti-GFAP antibody at 4°C overnight. The second antibody was Goat anti-Rat IgG H&L and Goat anti-rabbit IgG H&L (**Table 1**). The duration of DAPI staining was 10 min. Slices were sealed with Antifade Mounting Medium (Beyotime Biotechnology, P0126) and kept in dark until image capture.

#### BrdU and Iba1 or DCX Double Immuno-Staining

Slices were preprocessed with antigen retrieval solution (1×; Beyotime Biotechnology, P0092) at 80°C for 30 min. After DNA was denatured with HCl at room temperature, slices were incubated with anti-BrdU antibody and anti-Iba1 antibody or anti-Doublecortin (DCX) antibody. The second antibodies were Goat anti-Rat IgG H&L and Goat anti-rabbit IgG H&L. DAPI was used for nucleus staining.

### Western Blot

The hippocampus was collected on ice and stored at −80°C. The total protein of hippocampus was homogenized with RIPA lysis buﬀer (P0013B, Beyotime Biotechnology, Shanghai, China) containing PMSF, and phosphatase inhibitor on ice following incubation for 30 min at 4°C. Hippocampal lysates were centrifuged at 10,000 ×g for 10 min at 4°C. Supernatant was collected and the protein concentration was determined by a Bicinchoninic acid Protein Assay kit (P0009, Beyotime, Shanghai, China). The loading buffer was used to adjust the protein concentration and 30 µg protein was loaded and separated by 10% sodium SDS–PAGE (Beyotime Biotechnology) before being transferred to a PVDF membrane (Millipore). After 1 h BSA blocking, membrane was incubated with rabbit anti-Iba1 antibody, rabbit anti-CX3CL1 antibody, rabbit anti-CX3CR1 antibody, anti-PKA antibody, rabbit anti-ErK antibody, rabbit anti-pErK, and rabbit anti-GAPDH. After being incubated with second antibody Goat Anti-Rabbit IgG H&L (HRP), membranes were washed with TBST 5 min for 5 times. The protein blots were visualization with chemiluminescent HRP substrate (P90720, Millipore Corporation, Burlington, MA) and detected by Molecular Imager ChemiDocTM XRS+ analysis system (BioRad Co., Hercules, CA). Image J was used for quantitative Western Blot analysis. All experimental were repeated four times.

## Statistical Analysis

All data were expressed as the mean ± SEM. All statistical analyses were performed using SPSS software package (IBM). The difference among three or more groups was analyzed by two-way repeated-measures analysis of variance (ANOVA) for statistical significance. Two-way ANOVA, paired and unpaired Student’s *t*-tests were also used for data analysis. Differences of *p *< 0.05 were considered statistically significant.

## Result

### Maternal Nicotine Exposure Alters Physical and Neurobehavioral Development of the Offspring

**Figure 1:**
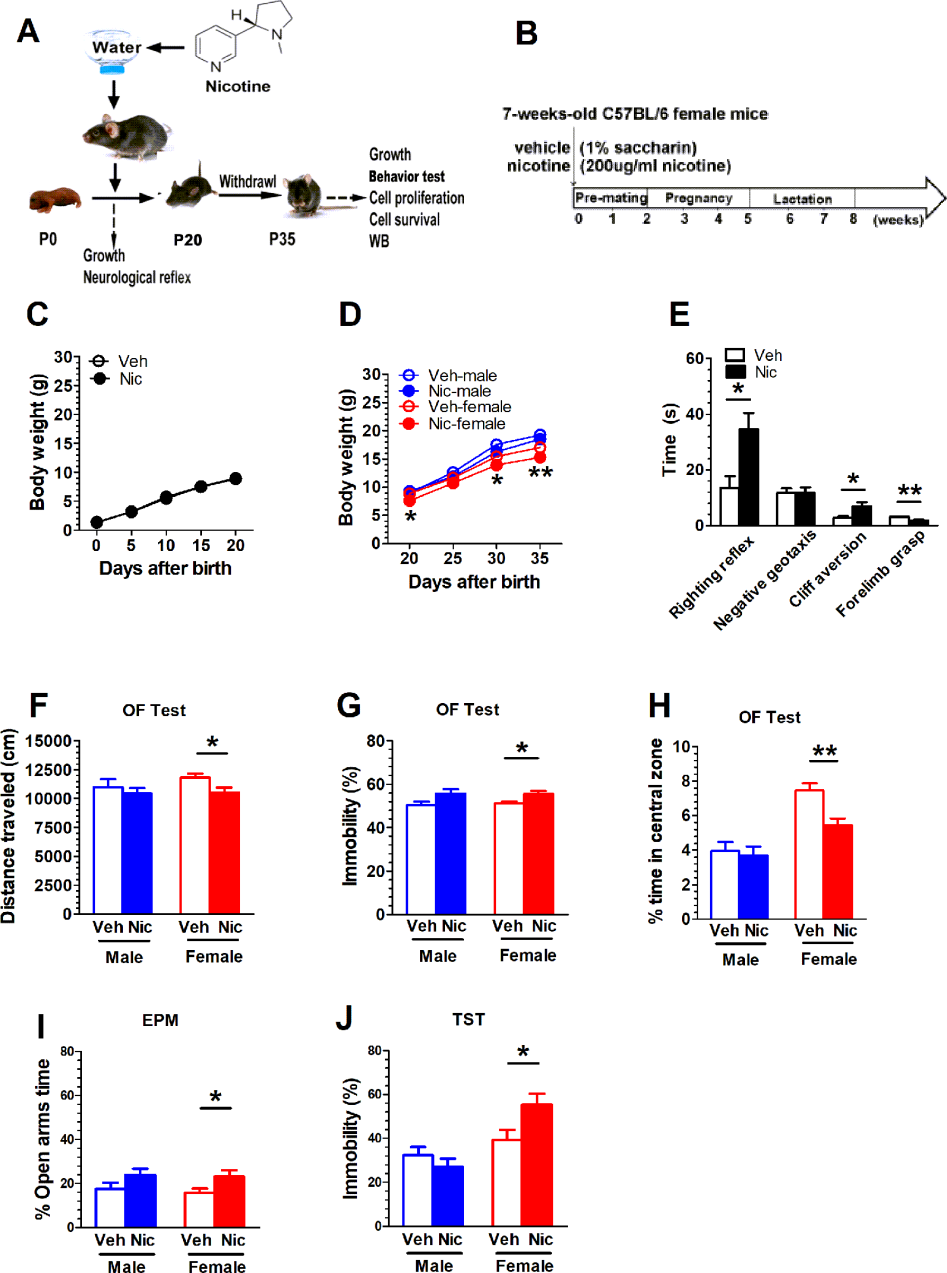
Maternal nicotine exposure alters female nicotine offspring locomotor and exploration behavior during adolescence. **(A, B)** show the experimental paradigm of maternal nicotine exposure and tests in the offspring. Mice were weaned on P20 and behavioral tests were conducted on P35. **(C)** There was no difference in body weight from P0 to P20 between nicotine offspring (n = 25) and vehicle offspring (n = 25). **(D)** Male mice presented no significant difference in body weight from P20 to P35 (vehicle: n = 13; nicotine: n = 12), but there was significant difference in female mice (vehicle: n = 12; nicotine: n = 13). **(E)** The time mice spent in cliff aversion or reverting to the prone position in nicotine offspring was significantly longer than that in vehicle offspring (n = 25). However, there was no significant difference in the time of turn 180° from head down to head up between the two groups (n = 25). Moreover, the time of forelimb grasp in nicotine offspring was significantly less than the vehicle offspring (n = 25). Open field test showed that female nicotine offspring presented a decrease in total distance traveled (n = 12) **(F)**, an increase in immobility (n = 12) **(G)** and a decrease in the time the open-field center area (n = 12) **(H)** male mice did not present a difference in behavioral outputs in open-field test. **(I)** The female offspring increased the time spent in the open arms of the EPM (n = 12), but no significant difference in male mice (n = 12). **(J)** The nicotine female offspring significantly increased the immobility time in comparison with the vehicle female mice (n = 12). But there was no statistical difference in male mice (n = 12) *p < 0.05, **p < 0.01.

**Figures 1A, B** show the experimental paradigm of maternal nicotine exposure and tests in the offspring. The cohort size of the offspring from female mice (n = 5) that experienced nicotine exposure (nicotine offspring, 6 ± 0.66) was lower than the cohort size of the offspring from female mice (n = 5) that received only vehicle treatment (vehicle offspring, 8 ± 0.89) (*t*(8) = 1.62, *p* = 0.14). There was no difference in body weight from P0 to P20 between nicotine offspring and vehicle offspring (**Figure 1C**). A two-way repeated measures ANOVA showed a significant difference in time (*F*(4,120) = 6.34, *p* = 0.02, n = 25), but not in group (*F*(1,48) = 0.81, *p* = 0.372, n = 25), time × group interaction(*F*(4,120) = 1.77, *p* = 0.19, n = 25; **Figure 1C**). The offspring genders were identified on P20. Similarly, male mice presented no significant difference in body weight from P20 to P35 *(F* (1,23) = 2.13, *p* = 0.16, n = 12-13), but there was a significant difference in female mice (*F* (1,23) = 7.28, *p* = 0.013, n = 12-13; **Figure 1D**). The physical development in the two groups were significantly different: hair growth (Veh, 5.60 ± 0.24, Nic, 7.60 ± 0.40; *t*(8) = 4.26, *p* = 0.0027, n = 5), pinna detachment (Veh, 4.20 ± 0.20, Nic, 5.60 ± 0.24; *t*(8) = 4.43, *p* = 0.0022, n = 5), incisor eruption (Veh, 10.60 ± 0.24, Nic, 12.20± 0.20; *t*(8) = 5.06, *p* = 0.001, n = 5), and eye-opening (Veh, 14.00 ± 0.32, Nic, 16.00 ± 0.32; *t*(8) = 4.47, *p* = 0.0021, n = 5) significantly later than those in vehicle mice (**Table 2**). Neurodevelopmental retardation in prelactation was evaluated by various reflexive measures. The nicotine mice were significantly later than the vehicle group in terms of air righting reflex (Veh, 9.40 ± 0.24, Nic,13.60 ± 0.24; *t*(8) = 12.12, *p* < 0.0001, n = 5), auditory startle (Veh, 12.60 ± 0.24, Nic, 15.00 ± 0.32; *t*(8) = 6, *p* = 0.0003, n = 5), and olfactory reflex (Veh, 12.20 ± 0.20, Nic,14.20 ± 0.49; *t*(8) = 3.78, *p* = 0.0054, n = 5) (**Table 2**). As shown in **Figure 1E**, the time mice spent reverting to the prone position in nicotine offspring was significantly longer than that in vehicle offspring (*t*(48) = 2.57, *p* = 0.013, n = 25). Similarly, the time mice spent in cliff aversion in nicotine offspring was significantly longer than that in vehicle offspring (*t*(48) = 2.57, *p* = 0.013, n = 25). However, there was no significant difference in the time of turn 180° from head down to head up between the two groups (*t*(48) = 0.02, p = 0.99, n = 25). Moreover, the time of forelimb grasp in nicotine offspring was significantly shorter than the vehicle offspring (*t*(48) = 3.49, *p* = 0.001, n = 25).

**Table 2:** Physiological development index in each different group.

Observation	Vehicle offspring	Nicotine offspring
Hair growth	5.60 ± 0.24	7.60 ± 0.40^**^
Pinna detachment	4.20 ± 0.20	5.60 ± 0.24^**^
Incisor eruption	10.60 ± 0.24	12.20 ± 0.20^***^
Eye-opening	14.00 ± 0.32	16.00 ± 0.32^**^
Auditory startle	12.60 ± 0.24	15.00 ± 0.32^***^
Olfactory reflex	12.20 ± 0.20	14.20 ± 0.49^**^
Air righting	9.40 ± 0.24	13.60 ± 0.24^***^

**P < 0.01，***P < 0.001.

### Maternal Nicotine Exposure Alters Female Nicotine Offspring Locomotor and Exploration Behavior During Adolescence

Mice were weaned on P20 and behavioral tests were conducted on P35. Open field test showed that female nicotine offspring presented a decrease in total distance traveled (*t*(22) = 2.35, *p* = 0.03, n = 12; **Figure 1F**), A two-way ANOVA did not reveal treatment × gender interaction difference in total distance (*F*(1,44) = 0.56, *p* = 0.46, n = 12), immobility (F(1.44) = 0.07, *p* = 0.79, n = 12), and in center area(F(1,44) = 0.56 *p* = 0.46, n = 12). Female nicotine offspring presented an increase in immobility (*t*(22) = 2.63, *p* = 0.02, n = 12; **Figure 1G**) and a decrease in the time spent in the open-field center area (*t*(22) = 3.46, *p* = 0.0023, n = 12; **Figure 1H**). Male mice did not present a difference in behavioral outputs in the open-field test. Both male and female nicotine offspring presented an increase in the time spent in the open arms of the EPM. There was significant difference in female offspring (*t*(22) = 2.21, *p* = 0.04, n = 12);, but no significant difference in male mice (*t*(22) = 1.562, *p* = 0.13, n = 12; **Figure 1I**). During the Tail suspension test, the female nicotine offspring presented an increase in the immobility time in comparison with the vehicle cohort (*t*(22) = 2.41, *p* = 0.02, n = 12; **Figure 1J**). No difference was observed in male mice (*t*(22) = 1.012, *p* = 0.32, n = 12; **Figure 1J**).

### Prenatal and Lactation Nicotine Exposure Facilitates the Proliferation of Newborn Neural Cell in Hippocampal DG of the Offspring

**Figure 2:**
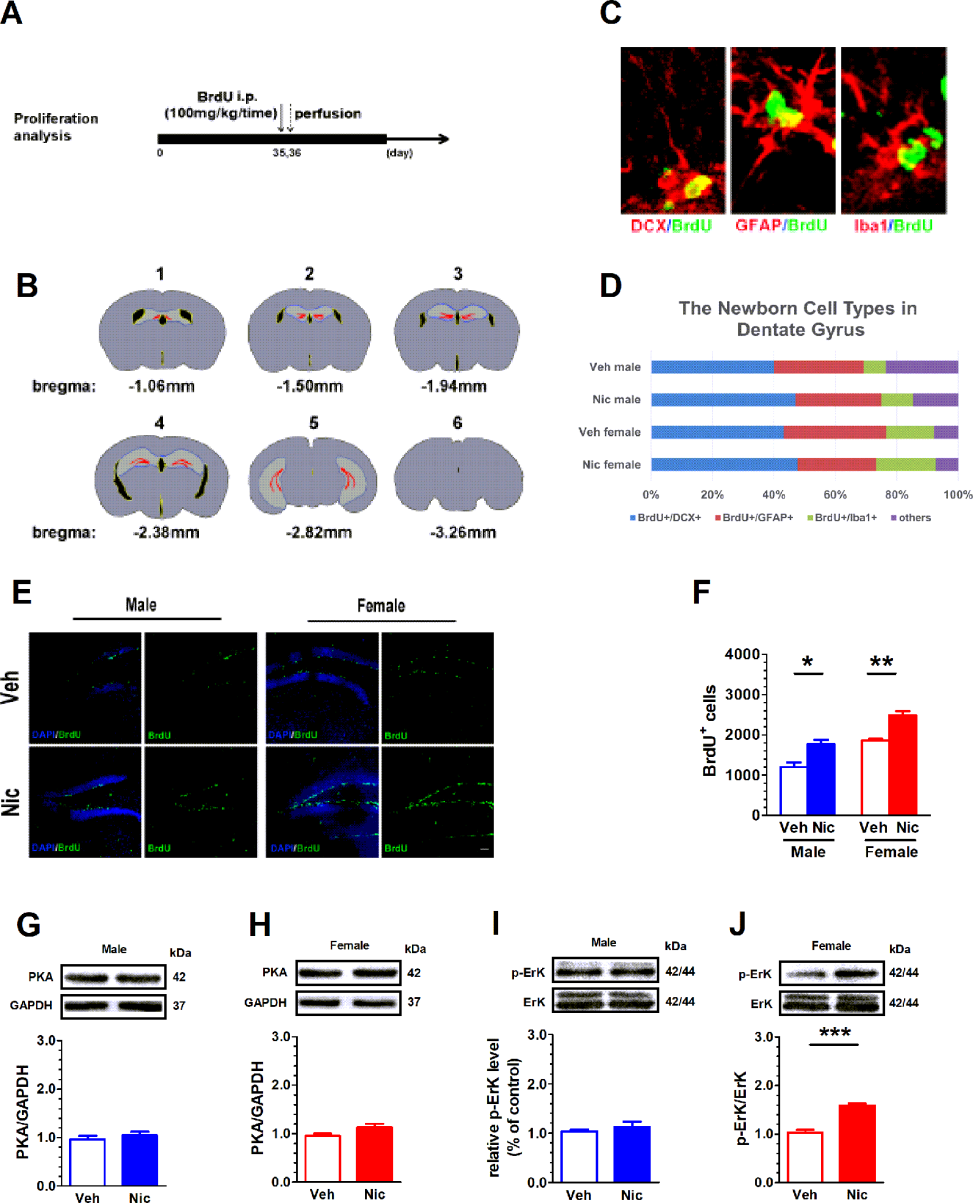
Prenatal and lactation nicotine exposure facilitates the proliferation of newborn neural cell in hippocampal dentate gyrus (DG) of the offspring. BrdU staining was used to study neurogenesis and proliferation in hippocampal DG. **(A)** shows the experimental paradigm and **(B)** shows the DG location across coronal sections. **(C)** The BrdU was colabeled with GFAP, Iba1, and DCX in the adult DG. **(D)** Percentage of newborn cells of different types in dentate gyrus. **(E)** Shows DG BrdU staining in vehicle and nicotine offspring (n = 4). **(F)** The number of BrdU^+^ cells in the DG region was elevated in both male and female nicotine offspring (n = 4). Neither male **(G)** nor female **(H)** offspring (n = 4) presented a difference in PKA between nicotine and vehicle treatment. pErK/ErK ratio was elevated in female **(J)**, but not in male **(I)** nicotine offspring. *p < 0.05, **p < 0.01, ***p < 0.001. Scale bar = 50 µm.

BrdU staining was used to study neurogenesis and proliferation in hippocampal DG. **Figure 2A** shows the experimental paradigm and **Figure 2B** shows the DG location across coronal sections. The BrdU was colabeled with GFAP, an astrocyte marker protein, Iba1, a microglia marker protein, and DCX, a microtubule-associated phosphoprotein utilized as a marker of newly born neurons in the adult DG (**Figure 2C**). We analyzed the percentage of various cells in total BrdU positive cells. BrdU^+^/DCX^+^ positive cells accounted for the majority of all newborn cells (**Figure 2D**). **Figure 2E** shows DG BrdU staining in vehicle and nicotine offspring. The number of BrdU^+^ cells in the DG region was elevated in both male (*t*(6) = 3.54, *p* = 0.012, n = 4) and female nicotine offspring (*t*(6) = 5.616, *p* = 0.0014, n = 4). A two-way ANOVA showed a significant difference in treatment (*F*(1,6) = 4.89, *p* = 0.046, n = 4), but not in sex (*F*(1,6) = 2.02, *p* = 0.18, n = 4; **Figure 2F**). PKA and p-ERK modulate cell proliferation. Neither male nor female offspring presented a difference in PKA between nicotine and vehicle treatment (**Figures 2G, H**). p-ERK protein level was elevated only in female nicotine offspring (*t*(6) = 8.254, *p* = 0.002, n = 4; **Figure 2J**), but not male offspring (*t*(6) = 0.72, *p* = 0.49, n = 4; **Figure 2I**).

### Prenatal and Lactation Nicotine Exposure Reduces Survival of Newborn Cells in Hippocampal DG SGZ in Female

**Figure 3:**
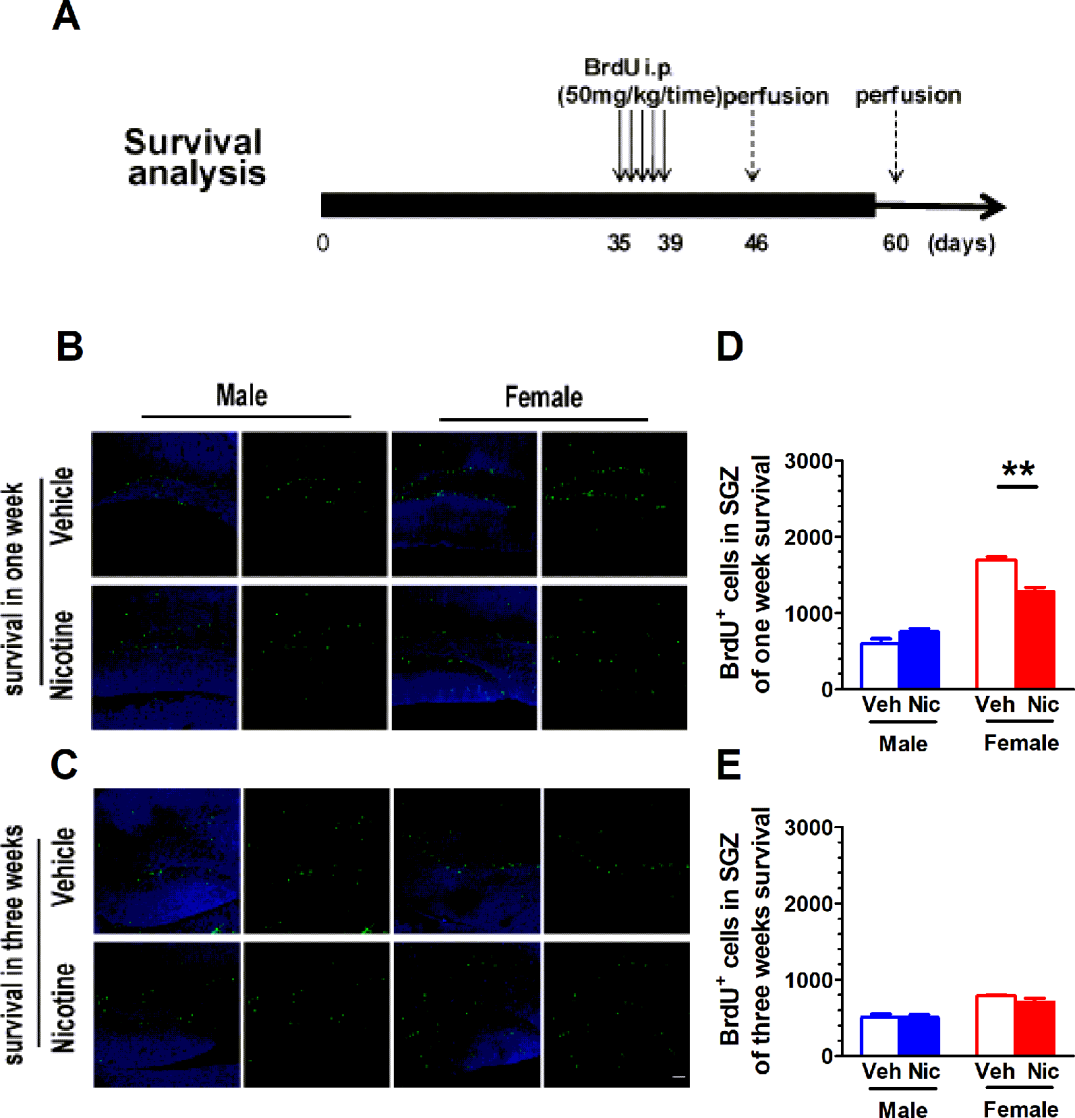
Prenatal and lactation nicotine exposure reduces survival of newborn cells in hippocampal dentate gyrus (DG) SGZ only in female offspring. **(A)** shows the paradigm for survival analysis. The BrdU was injected once a day starting from P35 for five consecutive days. BrdU^+^ cells were examined on day 7 (one-week survival) and day 21 (three-week survival) after the last BrdU injection. **(B)** Representative BrdU^+^ staining image on day 7. The newly proliferated neural cells in nicotine offspring (n = 4) were significantly less than those in vehicle offspring (n = 4) in the female group. **(C)** Representative BrdU^+^ staining image on day 21. There was no difference in the newly proliferated neural cells in both male and female groups (n = 4). **(D)** BrdU^+^ cells throughout the SGZ were quantified in offspring of one-week survival and three-week survival **(E)**. **p < 0.01. Scale bar = 50 µm.

**Figure 3A** shows the paradigm for survival analysis. The BrdU was injected once a day starting from P35 for five consecutive days. BrdU^+^ cells were examined on day 7 (one-week survival) and day 21 (three-week survival) after the last BrdU injection. The number of BrdU^+^ cells decreased with age (**Figures 3B, C**). On P35, the newly proliferated neural cells in nicotine offspring were significantly less than those in vehicle offspring in females (male: *t*(6) = 2.01, *p* = 0.09, n = 4; female: *t*(6) = 5.72, *p* = 0.0012, n = 4; **Figure 3D**). Three weeks after the last injection of BrdU, most of the newly proliferated neural cells died, and the survival rate leveled off. There was no significant difference in male (*t*(6) = 0.06, *p* = 0.95, n = 4) and female offspring (*t*(6) = 1.60, *p* = 0.16, n = 4; **Figure 3E**).

### Prenatal and Lactation Nicotine Exposure Affects the Incidence of Disarrayed and the Number of Newborn Neurons

**Figure 4:**
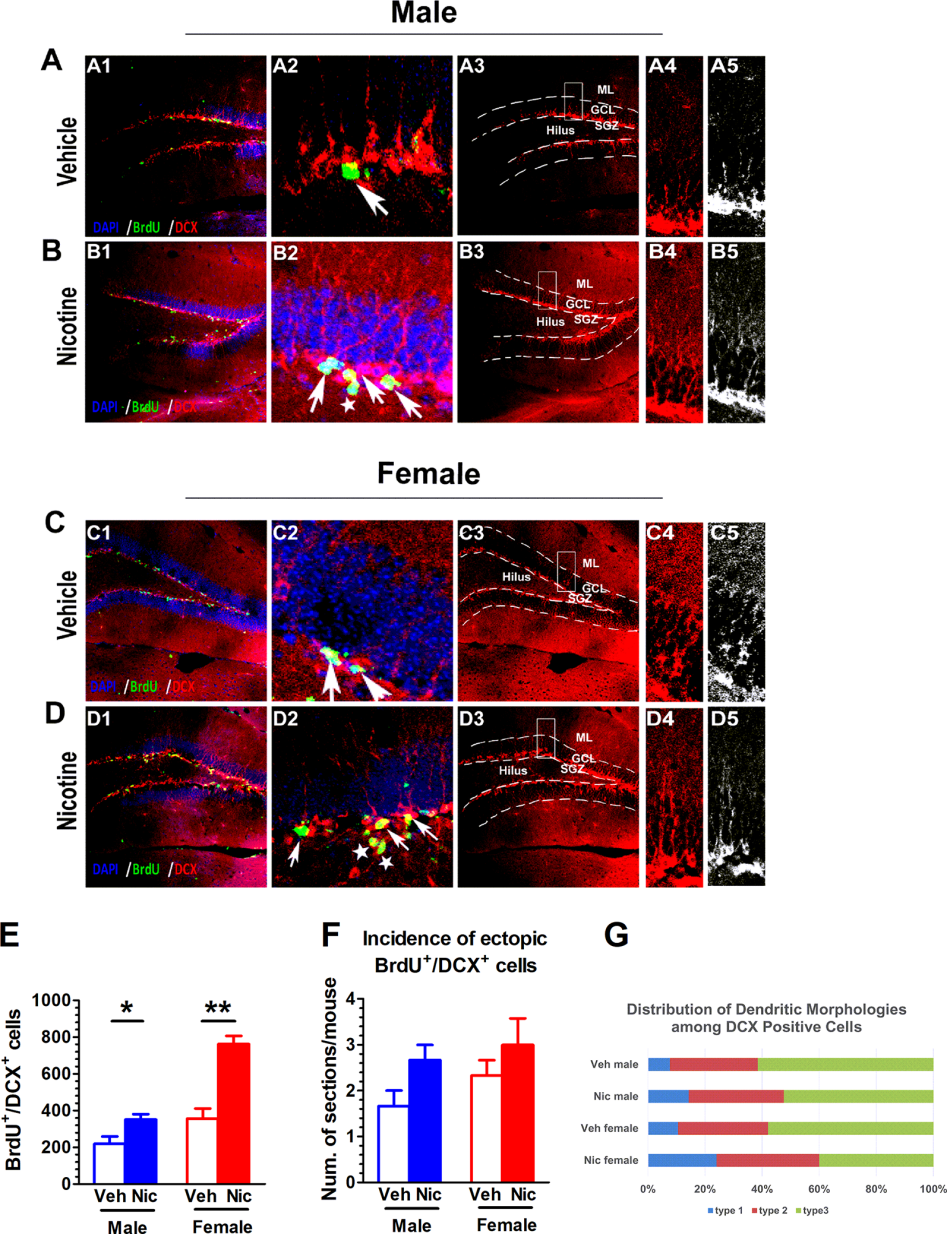
Prenatal and lactation nicotine exposure affects the incidence of disarrayed and the number of newborn neurons. **(A**–**D)** DCX^+^ staining was only observed in immature neuroblasts and progenitor cells. Most DCX^+^ cells of female nicotine offspring presented high diameter dendrite branching in the molecular layer (ML) in the postmitotic phase **(D)**. The DCX^+^ cells in vehicle offspring with delicate dendritic tree branching in the granular cell layer (GCL) **(A**, **C)**. This phenomenon was not observed in male nicotine offspring **(B)**. **(E)** Both male and female nicotine offspring (n = 4) presented an increase in the number of BrdU^+^/DCX^+^ cells as compared to vehicle offspring on P35. **(F)** The DCX^+^ young newborn neurons in the vehicle offspring (n = 4) were located in the germinative SGZ, while the nicotine offspring (n = 4) had higher incidence migrating from the SGZ to the granule cell layer (GCL), but there was no difference in cell number in the brain section. **(G)** Distribution of Dendritic Morphologies among DCX^+^/BrdU^+^ Cells. *p < 0.05, **p < 0.01. Scale bar = 50 µm.

DCX is an immature neuronal marker and BrdU identifies proliferating cells. DCX can only be observed in immature neuroblasts and progenitor cells. We categorized DCX positive cells into three categories according to the presence and the shape of apical dendrites and their presumed sequential order refereeing to Plumpe’s report (Plumpe et al., 2006). Type 1 were cells with no or very short processes. Type 2 cells were with processes of intermediate length and immature morphology, and Type 3 cells were with a more mature appearance. The distribution to these three types of DCX-positive cells was quantified (**Figure 4G**). Most DCX^+^ cells of female nicotine offspring presented high diameter dendrite branching in the molecular layer (ML) in the postmitotic phase (**Figure 4D**). The DCX^+^ cells in vehicle offspring had delicate dendritic tree branching in the granular cell layer (**Figures 4A, C**). Interestingly, this phenomenon was not observed in male nicotine offspring (**Figure 4B**). BrdU and DCX staining was used to identify neuroblasts in the hippocampal SGZ on P35. Both male and female nicotine offspring presented an increase in the number of BrdU^+^/DCX^+^ cells as compared to vehicle offspring (male: *t*(6) = 2.63, *p* = 0.039, n = 4; female: *t*(6) = 5.69, *p* = 0.0013, n = 4; **Figure 4E**). BrdU^+^/DCX^+^ cells, as displaced newborn cells that travelled into the hilus, showed aberrant neurogenesis (Jonsson et al., 1997; Parent et al., 1997; McCloskey et al., 2006; Plumpe et al., 2006; McClain et al., 2014).

The DCX^+^ young newborn neurons in the vehicle offspring were located in the germinative SGZ, while the nicotine offspring had higher incidence of migration from the SGZ to the granule cell layer, which revealed ectopic neurogenesis. There was no difference in cell number in the brain section (male: *t*(6) = 1.73, *p* = 0.13, n = 4; female: *t*(6) = 1.57, *p* = 0.17; n = 4; **Figure 4F**).

### Nicotine Offspring Present an Increase in Microglial Markers

**Figure 5:**
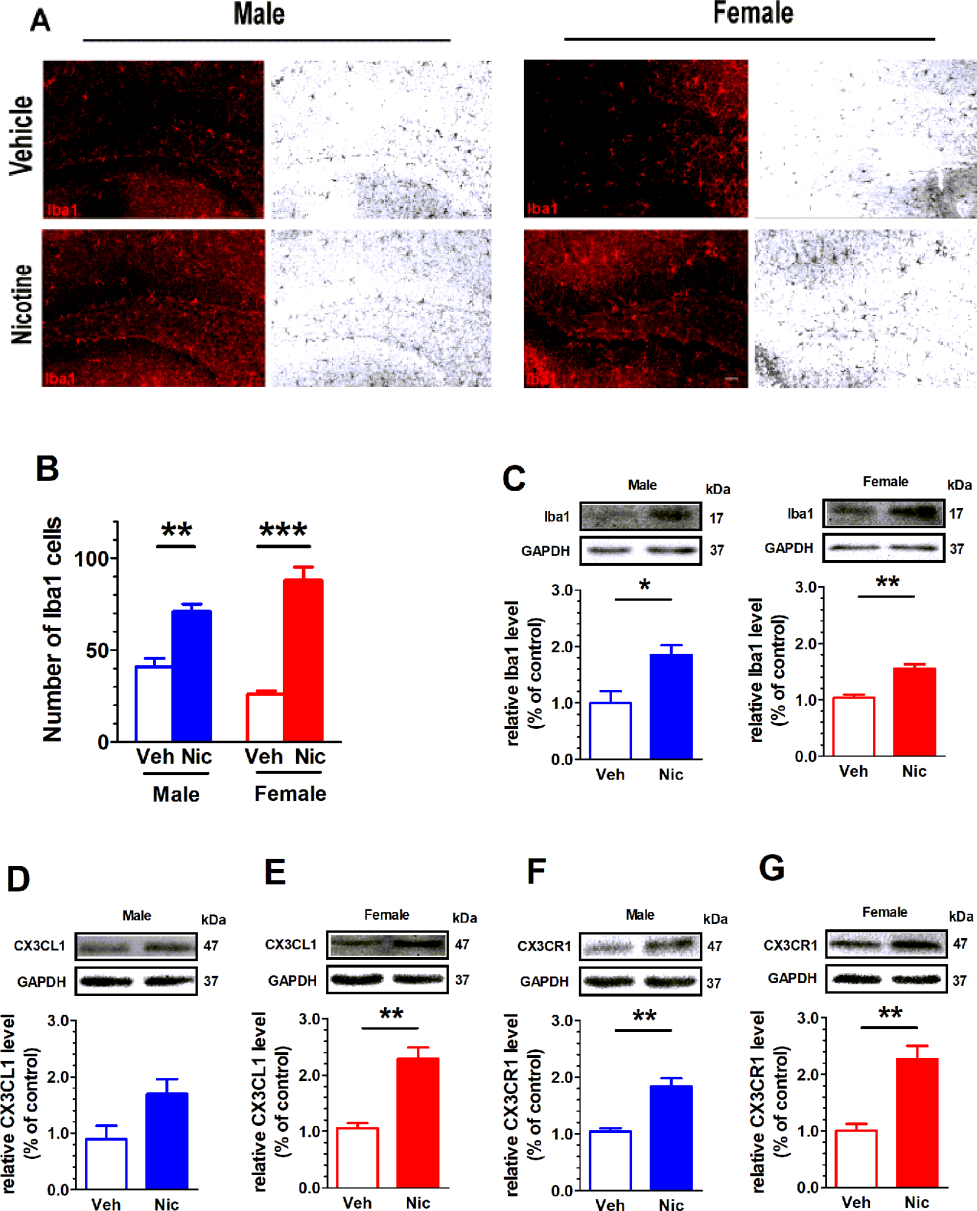
Nicotine offspring present an increase in microglial markers. **(A)** Hippocampal Iba1^+^ staining. Both male and female nicotine offspring (n = 4) presented an increase in the density of hippocampal Iba1^+^ fluorescent staining **(B)** and protein bands **(C)** as compared to vehicle offspring (n = 4). **(D, E)** CX3CL1 protein level was significantly increased in female nicotine offspring (n = 4), but not male nicotine offspring (n = 4), as compared to vehicle control (n = 4). **(F, G)** CX3CR1 protein level was significantly higher in both female nicotine offspring (n = 4) and male nicotine offspring (n = 4) than that in control subjects. *p < 0.05, **p < 0.01, ***p < 0.001. Scale bar = 50 μm.

The expression of Microglial marker Iba1 in the hippocampus was examined with immunofluorescent staining and Western blot. **Figure 5A** shows the hippocampal Iba1^+^ staining. Both male and female nicotine offspring presented an increase in the density of hippocampal Iba1^+^ fluorescent staining (male: *t*(6) = 4.87, *p* = 0.0028, n = 4; female: *t*(6) = 8.39, *p* = 0.0002, n = 4; **Figure 5B**) and protein bands (male: *t*(6) = 3.22, *p* = 0.03, n = 4; female: *t*(6) = 5.42, *p* = 0.002, n = 4; **Figure 5C**) as compared to vehicle offspring. To further confirm the upregulation of hippocampal microglia, the microglia-specific CX3CR1 protein was tested. Consistently, an increase in CX3CR1 protein level was observed in both male (*t*(6) = 5.35, *p* = 0.002, n = 4) and female (*t*(6) = 4.83, *p* = 0.003, n = 4) nicotine offspring as compared to that in vehicle control mice (**Figures 5F, G**). Interestingly, CX3CL1 protein level was only elevated in female nicotine offspring (*t*(6) = 5.65, *p* = 0.013, n = 4), and no significant difference was observed in male nicotine offspring (*t*(6) = 2.28, *p* = 0.063, n = 4) as compared to vehicle control (**Figures 5D, E**).

## Discussion

The impact of maternal nicotine exposure on the behavioral performance and neurobiological characteristics during the adolescent stage remain elusive. In this study, we explored maternal nicotine exposure during prepregnancy, pregnancy, and lactation periods on the behavioral outputs and hippocampal neurogenesis of the offspring during the adolescent stage. Besides behavioral change, nicotine offspring presented an increase in cell proliferation in hippocampal DG, but cell survival was decreased in female offspring. Nicotine offspring presented an increase in the occurrence of ectopic of DCX^+^ cells and Iba1 expression. Our data revealed that maternal nicotine exposure affect both hippocampal neurogenesis and microglial activity.

It is well established that maternal cigarette smoking results in intrauterine growth restriction and postnatal development (England et al., 2001). Previous studies have demonstrated that prenatal nicotine exposure is associated with the occurrence of attention-deficit/hyperactivity disorder (ADHD), anxiety, and depression-like behavior in adolescent and adult rats (Alkam et al., 2013). Consistently, our data reveal that maternal nicotine exposure across the prepregnancy, pregnancy, and lactation stages decreases the locomotor activity in female offspring during the adolescent stage. The horizontal locomotor activity was recorded by an open-field test. Only female nicotine offspring present a decrease in distance traveled and an increase in immobility. Moreover, female nicotine offspring spent less time in the central area of the open field. It suggests that maternal nicotine exposure produces distinct effects on male and female offspring. In line with these, other studies also show that prenatal nicotine exposure in rodents causes postnatal hyperactivity, cognitive impairment, increased anxiety, somatosensory deficits, persistent neurochemical alterations, changes in sensitivity to nicotine, alterations in nicotine self-administration and altered patterns of neural cell survival, and synaptogenesis (Dwyer et al., 2009).

Nicotine is the main toxic substance in tobacco. Nicotine *via* oral administration (in tap water) has been used in a variety of experiment including addiction, withdrawal, and toxicity on the offspring (Grabus et al., 2005; Matta et al., 2007; Zhao-Shea et al., 2015; Mojica et al., 2018). Nicotine treated mice with 200 μg/ml in drinking had at least 300 ng/ml serum cotinine, consistent with concentrations achieved by heavy smokers (Lawson et al., 1998; Heath et al., 2010; Mojica et al., 2018). Moreover, nicotine level in breast milk is even higher than that in the blood, primarily due to the partitioning of nicotine into the high-lipid-containing, more acidic milk when it was administrated *via* drinking water (Matta et al., 2007).

Female mice that exposed to nicotine presented a trend of producing less offspring, but no statistical significance was observed. There was no difference in the growth of body weight. However, early physical development index and early neurobehavioral developmental index of offspring showed delayed development in children of the nicotine group. The reflex righting time of nicotine offspring was significantly longer than those of the vehicle group. Nicotine exposure in utero or early postpartum impairs limbic and cortical neuronal development, mood, and cognitive function (Alkam et al., 2013). The data suggest that the early stage of neural development may be delayed in the nicotine offspring.

Maternal nicotine exposure may induce neurobiological disorders in the offspring, for example, ADHD, learning, and memory problems. The neural development of the hippocampus undergoes a wide range of neurogenesis, including neural maturation, synaptogenesis of neurons, and synaptic stability (Wang and Gondre-Lewis, 2013). The generation, maturation and integration of new neurons in adult hippocampus are crucial for hippocampal function, including learning and memory. In this study, the effect of maternal nicotine exposure during prepregnancy, pregnancy, and lactation on the neurogenesis in the offspring was observed by BrdU labeling of newborn neural cells. Interestingly, both male and female nicotine offspring present a significant increase in the number of proliferated neural or newborn neurons as compared to vehicle group.

However, the migration rate of newborn neurons of nicotine offspring was significantly higher than that of the vehicle group, and the synaptic branches were reduced. The 1-week survival rate of proliferated neural cells was significantly lower than that of the vehicle group in the female offspring, suggesting that an increase of newborn neural cells in the adolescent offspring is not beneficial to the survival of newborn neural cells. Moreover, the morphological change that BrdU^+^/DCX^+^ cells, as displaced newborn cells that travelled into the hilus, showed aberrant neurogenesis in the nicotine offspring further supports the view that maternal nicotine exposure produces a long-term effect on neurogenesis and structure modification. Nicotine precipitated changes in neuronal growth, survival, and synaptic plasticity, which may be an underlying mechanism of behavioral abnormalities as observed in nicotine offspring.

Microglia have a variety of functions, for example, supporting central nervous system development and synaptic formation, maintaining homeostasis, immune response to infectious agents, and involvement in adult neurogenesis, neuroinflammation, degenerative disease, stroke, trauma, and regeneration (Lannes et al., 2017). Our study reveals that microglial marker protein Iba1 was elevated in nicotine offspring. Microglia play an important role in neuroinflammation and dysfunction. Microglia activation is accompanied by changes in morphology and signal transduction (Ben Haim et al., 2015). Such changes in microglia may affect synaptic formation and lead to defects in synaptic branches, which may lead to cognitive impairment and neurodegenerative diseases (Chung et al., 2015). Microglia stretch and contract to constantly monitor their local environment, send signals to neurons, and actively maintain synaptic health. The increase in Iba1 expression may suggest the disruption of glia-neuron communication.

The early embryonic development of microglia originates from the 7.5 day of embryonic development and produces nuclear red cells and macrophages in the whole embryo. Macrophages enter the central nervous system before the blood-brain barrier is shut down on the 9th day of embryonic development. Immune cells gradually differentiate into microglia cells in the second week after birth (Thion et al., 2018). Maternal nicotine exposure may produce direct and indirect effects on microglial development.

CX3CL1 is a chemokine with a unique structure and is the only member of the CX3C chemokine family. CX3CR1 is mainly expressed in microglia. CX3CL1 may serve as a signal from neurons to glia, leading to microglial activation (Lindia et al., 2005). Our data revealed that Iba1, CX3CL, CX3CR1 were elevated in nicotine offspring, suggesting microglial hyperactivity. Activated microglia increases the release of a variety of proinflammatory cytokines and facilitates the occurrence of a number of behavioral and mental disorders (Nakayama et al., 2019). Our data reveal that maternal nicotine exposure produces a long-term effect on the microglial activity of the offspring. Activation of CX3CL1 signaling facilitates ErK phosphorylation. Our data revealed an increase in CX3CL1 protein level and p-Erk/Erk ratio in female nicotine offspring.

Nicotine replacement therapy (NRT) has been developed as a pharmacotherapy for smoking cessation and is considered to be a safer alternative than smoking for women during pregnancy. Smoking cessation programs based on behavioral therapy, which are implemented during pregnancy, have been shown to reduce the incidence of low birth weight and preterm birth (Lumley et al., 2004). As a result, we found changes in behavior and neurogenic/microglial markers examined during withdrawal in adolescent offspring after maternal nicotine exposure. It suggests that early life nicotine exposure not only retards somatic development such as physiological index and neurobehavioral developmental index, but also causes anxiety-like behavior alteration after withdrawal. Anxiety-like behaviors can be evaluated by open field, EPM, and tail suspension test in rodents. The EPM test is based on a rodent’s instinct to avoid open spaces and heights. The fear reaction in mice shows up in their tendency to stay in the closed arms of the maze and decreased motor activity. Their exploratory activity is assessed by measuring the time spent in the open arms. In the tail suspension test, the mice tried to escape but could not escape after hanging their tails, so they gave up their struggle and entered a special state of depression and immobility. The effects of early life nicotine exposure combination with deprivation afterwards on anxiety levels were investigated extensively in our study and our data indicate that maternal nicotine exposure impacts more on female offspring and female offspring present anxiety- and depression-like behaviors. Future study needs to be performed to examining how nicotine-induced change of neurogenesis and microglial effects on cognitive behaviors (e.g., novel object recognition and spatial memory) in offspring will further uncover the mechanism of nicotine toxicity in the hippocampus. However, our data provide additional evidence to support the negative effect of maternal NRT. In pregnant women who smoke or use NRT, nicotine crosses the placenta, concentrates in fetal blood and amniotic fluid, and is detectable in breast milk during lactation (Luck and Nau, 1987). Therefore, maternal NRT results in both fetal and neonatal exposure to nicotine.

This study demonstrates that maternal nicotine exposure in prepregnancy, pregnancy, and lactation stages impairs the behavioral performance, hippocampal neurogenesis, and microglial activation in the offspring. Our data support the view that maternal nicotine exposure produces an adverse effect on the offspring and is detrimental to the naturally occurring neurogenetic processes in the adult brain (Ngwenya and Danzer, 2018).

## Data Availability Statement

The datasets generated for this study are available on request to the corresponding author.

## Ethics Statement

The animal study was reviewed and approved by The Institutional Animal Care and Use Committee at Medical School of Anhui University of Science and Technology.

## Author Contributions

XT conceived and designed this experiment. FL, GP, DW, YH, ZX, SX, and BL collected data and drafted this manuscript. JL analyzed the data. LZ and QL performed the experiment and acquired data for the revision of this work. Y-MZ reviewed and edited the draft during revision.

## Funding

This work was supported by the China National Natural Science Foundation (81471161), Top Talent Projects of Anhui Department of Education (gxbjZD16), Quality Projects for Higher Education (2016ckjh074) and Graduate Student Innovation Fund (2017CX2115).

## Conflict of Interest

The authors declare that the research was conducted in the absence of any commercial or financial relationships that could be construed as a potential conflict of interest.
