# Supplementary material for: Maternal Nicotine Exposure During Gestation and Lactation Period Affects Behavior and Hippocampal Neurogenesis in Mouse Offspring
Source: Front Pharmacol. 2020 Jan 22;10:1569. doi: 10.3389/fphar.2019.01569 (PMC6987079; doi:10.3389/fphar.2019.01569)
Supplement: Supplementary file 1 [file Table_1.docx]

| Supplementary Table 1. Sample size | | | | | |  |
| --- | --- | --- | --- | --- | --- | --- |
| Vehicle | Before weaning |  | Body weight | n=25 |  |  |
|  |  |  | Developmental index (litter) | n=5 |  |  |
|  |  |  | Neuroreflex development | n=25 |  |  |
|  | After weaning | Male | Elevated plus maze | n=12 |  |  |
|  |  |  | Tail suspended | n=12 |  |  |
|  |  |  | Open-field | n=12 |  |  |
|  |  |  | Immunofluorescence  staining | Proliferation | Survival | |
|  |  |  |  |  | One week | Three weeks |
|  |  |  |  | n=4 | n=4 | n=4 |
|  |  |  | Western Blot | n=4 |  |  |
|  |  | Female | Elevated plus maze | n=12 |  |  |
|  |  |  | Tail suspended | n=12 |  |  |
|  |  |  | Open-field | n=12 |  |  |
|  |  |  | Immunofluorescence  staining | Proliferation | Survival | |
|  |  |  |  |  | One week | Three weeks |
|  |  |  |  | n=4 | n=4 | n=4 |
|  |  |  | Western Blot | n=4 |  |  |
| Nicotine | Before weaning |  | Body weight | n=25 |  |  |
|  |  |  | Developmental index (litter) | n=5 |  |  |
|  |  |  | Neuroreflex development | n=25 |  |  |
|  | After weaning | Male | elevated plus maze | n=12 |  |  |
|  |  |  | Tail suspended | n=12 |  |  |
|  |  |  | Open-field | n=12 |  |  |
|  |  |  | Immunofluorescence  staining | Proliferation | survival | |
|  |  |  |  |  | One week | Three weeks |
|  |  |  |  | n=4 | n=4 | n=4 |
|  |  |  | Western Blot | n=4 |  |  |
|  |  | Female | Elevated plus maze | n=12 |  |  |
|  |  |  | Tail suspended | n=12 |  |  |
|  |  |  | Open-field | n=12 |  |  |
|  |  |  | Immunofluorescence  staining | Proliferation | Survival | |
|  |  |  |  |  | One week | Three weeks |
|  |  |  |  | n=4 | n=4 | n=4 |
|  |  |  | Western Blot | n=4 |  |  |
